# Prognostic value of lung immune prognostic index in non-small cell lung cancer patients receiving immune checkpoint inhibitors: a meta-analysis

**DOI:** 10.3389/pore.2024.1611773

**Published:** 2024-06-20

**Authors:** Yi Wang, Yu Lei, Delai Zheng, Yanhui Yang, Lei Luo, Ji Li, Xiaoyang Xie

**Affiliations:** Department of Thoracic Surgery, The First People’s Hospital of Neijiang, Neijiang Affiliated Hospital of Chongqing Medical University, Neijiang, Sichuan, China

**Keywords:** lung immune prognostic index, non-small cell lung cancer, immune checkpoint inhibitor, prognosis, meta-analysis

## Abstract

**Background and Purpose:**

Until now, it has been difficult to accurately predict the efficacy of immunotherapy in patients with non-small cell lung cancer (NSCLC). A novel indicator, the lung immune prognostic index (LIPI), has shown relatively high prognostic value in patients with solid cancer. Therefore, this study aimed to further identify the association between LIPI and the survival of patients with NSCLC who receive immune checkpoint inhibitors (ICIs).

**Methods:**

Several electronic databases were searched for available publications up to April 23, 2023. Immunotherapy outcomes included overall survival (OS), progression-free survival (PFS), and hazard ratios (HRs) with 95% confidence intervals (CIs). Subgroup analysis based on the study design and comparison of the LIPI was conducted.

**Results:**

In this meta-analysis, 21 studies with 9,010 patients were included in this meta-analysis. The pooled results demonstrated that elevated LIPI was significantly associated with poor OS (HR = 2.50, 95% CI:2.09–2.99, *p* < 0.001) and PFS (HR = 1.77, 95% CI:1.64–1.91, *p* < 0.001). Subgroup analyses stratified by study design (retrospective vs. prospective) and comparison of LIPI (1 vs. 0, 2 vs. 0, 1–2 vs. 0, 2 vs. 1 vs. 0, 2 vs. 0–1 and 2 vs. 1) showed similar results.

**Conclusion:**

LIPI could serve as a novel and reliable prognostic factor in NSCLC treated with ICIs, and elevated LIPI predicts worse prognosis.

## Introduction

Lung cancer remains the most common malignancy and leading cause of tumor-related deaths worldwide [1, 2]. Non-small cell lung cancer (NSCLC) accounts for approximately 85% of all lung cancer cases [3]. Despite great advances in early screening, surgical techniques, and adjuvant therapies for NSCLC, the overall prognosis remains poor, representing a relatively high risk of recurrence and therapeutic resistance [4, 5]. In the last few years, immunotherapy has become an important treatment option for NSCLC, especially for patients with advanced-stage and metastatic NSCLC. Unfortunately, less than 20% of patients could benefit from immunotherapy [6].

In clinics, immune checkpoint inhibitors (ICIs), particularly anti-programmed death ligand 1 (PD-L1)/programmed death 1 (PD-1) antibodies, are widely used as first- or second-line treatments for metastatic/advanced NSCLC alone or in combination with chemotherapy. However, as mentioned above, the number of patients who benefit from ICIs is fairly limited [6]. Thus, accurate and effective indicators to predict the efficacy of ICIs are urgently needed to help select potential beneficiaries of ICIs. Overall, PD-L1 expression and tumor mutation burden (TMB) are the most commonly used biomarkers to select ICI-advantaged populations and predict prognosis. Nevertheless, the predictive effect of these two biomarkers on ICI efficacy is not satisfactory in clinical practice [7, 8]. Patients with high PD-L1 expression are more likely to experience better survival, but a subset of patients do not benefit from immunotherapy [9, 10]. Therefore, further exploration of effective predictive indicators for the prognosis of ICIs treated NSCLC is required.

Since the immune checkpoint pathway includes an important circulatory phase, changes in some parameters based on peripheral blood may be associated with the response to immunotherapy. Increasing evidence suggests that inflammatory responses play an essential role in the development and progression of cancers [11, 12]. The inflammatory process in the body is considered to be the immune resistance mechanism in cancer patients, which promotes the growth and spread of tumor cells and activates the carcinogenic signaling pathway [11, 12]. Some biomarkers, such as the neutrophil-to-lymphocyte ratio (NLR), derived neutrophil-to-lymphocyte ratio (dNLR), and platelet-to-lymphocyte ratio (PLR) have been used to detect inflammatory status and predict prognosis in various cancers, including NSCLC [11–14]. The lung immune prognostic index (LIPI) is a novel indicator based on a dNLR >3 and lactate dehydrogenase (LDH) > upper limit of normal range (ULN), which was first reported by Mezquita et al. [15]. Patients were divided into three groups based on the number of risk factors from the LIPI: low-risk, intermediate-risk, and high-risk groups with 0, 1, and 2 risk factors, respectively. Previous studies have revealed that pretreatment LIPI play a role in predicting the therapeutic outcomes of ICIs in patients with solid cancers. However, whether it can predict the prognosis of ICIs ICI-treated NSCLC remains unclear.

Therefore, this meta-analysis aimed to further identify the association between LIPI and survival of NSCLC patients receiving ICIs, which might contribute to the selection of an advantaged population and improvement of the therapeutic efficacy of immunotherapy among NSCLC patients.

## Materials and methods

The current meta-analysis was performed according to the Preferred Reporting Items for Systematic Reviews and Meta-Analyses 2020 [16].

### Literature search

The PubMed, EMBASE, Web of Science, and CNKI databases were searched from inception to April 23, 2023. The following terms were used for the search: PD-1, PD-L1, CTLA-4, ICIs, immune checkpoint inhibitor, lung, pulmonary, cancer, tumor, carcinoma, neoplasm, LIPI, lung immune prognostic index, survival, prognosis, and prognostic. The detailed search strategies were as follows: (PD-1 OR PD-L1 OR CTLA-4 OR ICIs OR immune checkpoint inhibitor) AND (lung OR pulmonary) AND (cancer OR tumor OR carcinoma OR neoplasm) AND (LIPI OR lung immune prognostic index) AND (survival OR prognosis OR prognostic). Free text and Medical Subject Headings terms were also applied. All the references cited in the included studies were reviewed.

### Inclusion criteria

Studies that met the following criteria were included:1) patients were pathologically diagnosed with primary NSCLC; 2) patients who received ICIs with or without other combined therapies such as chemotherapy; 3) LIPI score was assessed according to the dNLR values and LDH level before immunotherapy, and the association between LIPI and efficacy of immunotherapy was evaluated; 4) the overall survival (OS) and (or) progression-free survival (PFS) were defined as outcomes of immunotherapy; 5) hazard ratios (HRs) with 95% confidence intervals (CIs) for OS and PFS were directly reported in articles.

### Exclusion criteria

Studies that met any of the following criteria were excluded:1) low-quality studies; 2) letters, editorials, reviews, case reports, or animal trials; and 3) studies with insufficient or duplicated data.

### Data extraction

The following information was collected from the included studies: name of first author, publication year, country, study design (retrospective or prospective), sample size, TNM stage, pathological type, detailed drugs of ICIs, threshold and comparison of LIPI, endpoint, HR, and 95% CI.

### Quality assessment

Owing to the nature of the included studies, the Newcastle-Ottawa Scale (NOS) score system was used to evaluate the quality of the included studies. As mentioned above, only high-quality studies with an NOS score ≥6 were included.

The literature search, selection, information collection, and quality assessment were conducted by two authors independently and any disagreement was resolved by team discussion.

### Statistical analysis

All statistical analyses were performed using STATA 12.0. Heterogeneity between studies was evaluated using I^2^ statistics and Q test. If significant heterogeneity was observed (I^2^ > 50% and/or *p* < 0.1), the random-effects model was applied; otherwise, the fixed-effects model was used. HRs and 95% CIs were combined to evaluate the association between the LIPI, OS, and PFS. Subgroup analysis based on study design (retrospective vs. prospective) and comparison of LIPI (1 vs. 0, 2 vs. 0, 1–2 vs. 0, 2 vs. 1 vs. 0, 2 vs. 0–1 and 2 vs. 1) were conducted. Sensitivity analysis was conducted to detect the sources of heterogeneity and assess the stability of the overall results. Furthermore, Begg’s funnel plot and Egger’s test were conducted to detect publication bias, and significant publication bias was defined as *p* < 0.05 [17–19].

## Results

### Literature search and selection

**FIGURE 1:**
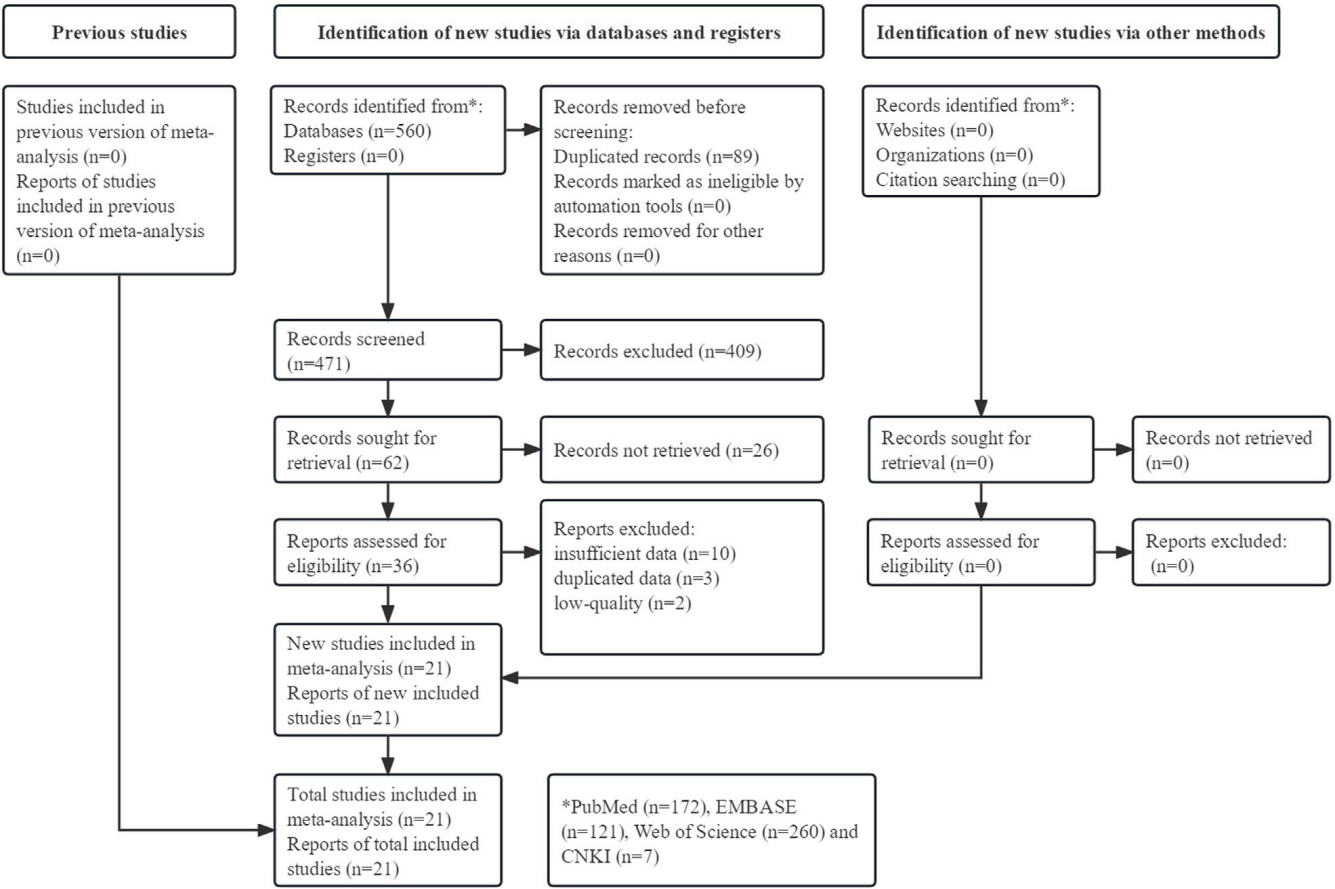
Prisma flow diagram of this meta-analysis.

The detailed process is illustrated in Figure 1. Initially, 560 records were searched from four databases and a total of 21 studies were included [15, 20–39].

### Basic characteristics of included studies

A total of 9,010 participants were enrolled in 21 studies published between 2018 and 2023. Most of the included studies were retrospective and focused on patients with advanced NSCLC. The sample size ranged from 51 to 1,489, and all studies applied the same definition of LIPI risk grading: LIPI 0, dNLR≤3 and LDH ≤ ULN; LIPI 1, dNLR >3 or LDH > ULN; and LIPI 2, dNLR >3 and LDH > ULN. Specific data are presented in Table 1.

**TABLE 1:** Basic characteristics of included studies.

Author	Year	Country	Study design	Sample size	TNM stage	Pathological type	ICIs	Threshold and comparison of LIPI	Endpoint	NOS
Mezquita [15]	2018	France	R	466	IIIB-IV	Mixed	Nivolumab, pembrolizumab, atezolizumab, durvalumab, and durvalumab-ipilimumab	0: dNLR≤3 and LDH ≤ ULN; 1: dNLR >3 or LDH > ULN; 2: dNLR >3 and LDH > ULN; 0 vs. 1 and 0 vs. 2	OS, PFS	7
Kazandjian [20]	2019	United States	P	1,368	IV	Mixed	NR	0: dNLR≤3 and LDH ≤ ULN; 1: dNLR >3 or LDH > ULN; 2: dNLR >3 and LDH > ULN; 0 vs. 2 and 1 vs. 2	OS, PFS	8
Ruiz-Bañobre [21]	2019	Spain	R	188	IIIB-IV	Mixed	Nivolumab	0: dNLR≤3 and LDH ≤ ULN; 1: dNLR >3 or LDH > ULN; 2: dNLR >3 and LDH > ULN; 0 vs. 1 vs. 2	OS, PFS	8
Sorich [22]	2019	Australia	P	1,489	Advanced	Mixed	Atezolizumab	0: dNLR≤3 and LDH ≤ ULN; 1: dNLR >3 or LDH > ULN; 2: dNLR >3 and LDH > ULN; 0 vs. 1 and 0 vs. 2	OS, PFS	7
Mazzaschi [23]	2020	Italy	P	109	IV	Mixed	NR	0: dNLR≤3 and LDH ≤ ULN; 1: dNLR >3 or LDH > ULN; 2: dNLR >3 and LDH > ULN; 0 vs. 1 vs. 2	OS, PFS	8
Wang [24]	2020	China	R	330	IIIB-IV	Mixed	Nivolumab, pembrolizumab, atezolizumab, and other PD-1/PD-L1 inhibitors	0: dNLR≤3 and LDH ≤ ULN; 1: dNLR >3 or LDH > ULN; 2: dNLR >3 and LDH > ULN; 0vs 1 and 0 vs. 2	OS, PFS	8
Ali [25]	2021	China	R	73	IV	Mixed	Pembrolizumab, nivolumab, camrelizumab and atezolizumab	0: dNLR≤3 and LDH ≤ ULN; 1: dNLR >3 or LDH > ULN; 2: dNLR >3 and LDH > ULN; 0 vs. 1–2	OS, PFS	6
Galland [27]	2021	France	R	231	NR	Adenocarcinoma	PD-1/PD-L1 inhibitors	0: dNLR≤3 and LDH ≤ ULN; 1: dNLR >3 or LDH > ULN; 2: dNLR >3 and LDH > ULN; 0 vs. 1–2	OS, PFS	6
Grosjean [28]	2021	Canada	R	327	I-IV	Mixed	Pembrolizumab	0: dNLR≤3 and LDH ≤ ULN; 1: dNLR >3 or LDH > ULN; 2: dNLR >3 and LDH > ULN; 0 vs. 1–2	OS	6
Hopkins [29]	2021	Australia	P	1,148	Advanced	Mixed	Atezolizumab	0: dNLR≤3 and LDH ≤ ULN; 1: dNLR >3 or LDH > ULN; 2: dNLR >3 and LDH > ULN; 0 vs. 1 and 0 vs. 2	OS, PFS	6
Mountzios [30]	2021	Greece	R	672	IV	Mixed	PD-L1 inhibitors	0: dNLR≤3 and LDH ≤ ULN; 1: dNLR >3 or LDH > ULN; 2: dNLR >3 and LDH > ULN; 0 vs. 1–2	OS, PFS	6
Chen [26]	2021	China	R	84	IIIB-IV	Mixed	PD-1/PD-L1 inhibitors	0: dNLR≤3 and LDH ≤ ULN; 1: dNLR >3 or LDH > ULN; 2: dNLR >3 and LDH > ULN; 0 vs. 1–2	OS, PFS	6
Chen [31]	2022	China	R	85	IV	Mixed	PD-1 inhibitors	0: dNLR≤3 and LDH ≤ ULN; 1: dNLR >3 or LDH > ULN; 2: dNLR >3 and LDH > ULN; 0 vs. 1–2	OS, PFS	6
De Giglio [32]	2022	Italy	R	182	IV	Mixed	NR	0: dNLR≤3 and LDH ≤ ULN; 1: dNLR >3 or LDH > ULN; 2: dNLR >3 and LDH > ULN; 0 vs. 1–2	OS	6
Holtzman [33]	2022	Israel	R	423	III-IV	Mixed	Pembrolizumab	0: dNLR≤3 and LDH ≤ ULN; 1: dNLR >3 or LDH > ULN; 2: dNLR >3 and LDH > ULN; 0 vs. 1–2	OS	6
Ortega-Franco [34]	2022	United Kingdom	R	113	III-IV	Mixed	Pembrolizumab	0: dNLR≤3 and LDH ≤ ULN; 1: dNLR >3 or LDH > ULN; 2: dNLR >3 and LDH > ULN; 0 vs. 1	OS, PFS	6
Tanaka [35]	2022	Japan	R	237	I-IV	Mixed	NR	0: dNLR≤3 and LDH ≤ ULN; 1: dNLR >3 or LDH > ULN; 2: dNLR >3 and LDH > ULN; 0–1 vs. 2	OS, PFS	6
Zhou J [36]	2022	China	R	51	IIIB-IV	Mixed	PD-1 inhibitors	0: dNLR≤3 and LDH ≤ ULN; 1: dNLR >3 or LDH > ULN; 2: dNLR >3 and LDH > ULN; 0 vs. 1–2	PFS	6
Zhou S [37]	2022	China	R	53	IV	Mixed	Pembrolizumab, nivolumab, sintilimab, camrelizumab, tislelizumab, and atezolizumab	0: dNLR≤3 and LDH ≤ ULN; 1: dNLR >3 or LDH > ULN; 2: dNLR >3 and LDH > ULN; 0–1 vs. 2	PFS	6
Zhou Y [38]	2022	China	R	86	I-IV	Mixed	Pembrolizumab, nivolumab and sindillimab	0: dNLR≤3 and LDH ≤ ULN; 1: dNLR >3 or LDH > ULN; 2: dNLR >3 and LDH > ULN; 0 vs. 1 and 0 vs. 2	PFS	7
Huang [39]	2023	China	R	147	IIIB-IV	Mixed	Nivolumab, pembrolizumab, atezolizumab, durvalumab, treprizumab, carrelizumab, sintilimab and tislelizumab	0: dNLR≤3 and LDH ≤ ULN; 1: dNLR >3 or LDH > ULN; 2: dNLR >3 and LDH > ULN; 0 vs. 2 and 1 vs. 2	PFS	7

ICIs: immune checkpoint inhibitors; LIPI: lung immune prognostic index; NOS: Newcastle-Ottawa Scale; R: retrospective; P: prospective; NR: not reported; PD-1: programmed death-1; PD-L1: programmed cell death 1 ligand 1; dNLR: derived neutrophil-to-lymphocyte ratio; ULN: upper limit of normal level; LDH: lactate dehydrogenase; OS: overall survival; PFS: progression-free survival.

### Association between LIPI and OS and PFS

**FIGURE 2:**
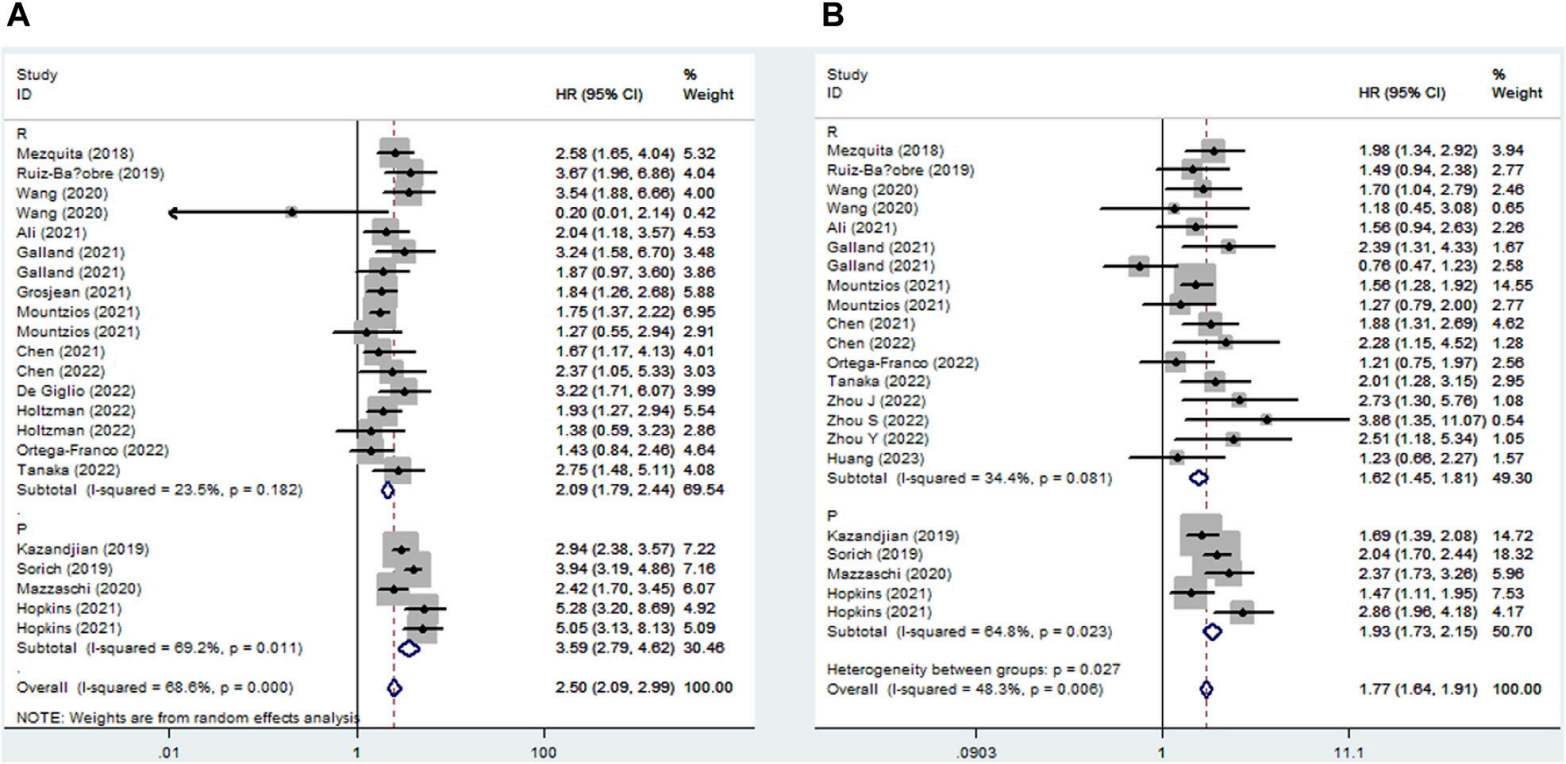
Subgroup analysis based on study design for the association between LIPI and overall survival **(A)** and progression-free survival **(B)** of non-small cell lung cancer patients receiving immune checkpoint inhibitors.

Seventeen studies explored the relationship between LIPI and OS in NSCLC patients receiving ICIs. The pooled results showed that elevated LIPI predicted poorer OS (HR = 2.50, 95% CI:2.09–2.99, *p* < 0.001; I^2^ = 68.6%, *p* < 0.001), and subgroup analysis based on the study design showed the same results (Figure 2A).

Eighteen studies identified a relationship between LIPI and PFS in immunotherapy-treated NSLC. The pooled results demonstrated that elevated LIPI was obviously associated with poor PFS (HR = 1.77, 95% CI:1.64–1.91, *p* < 0.001; I^2^ = 48.3%, *p* = 0.006), and subgroup analysis stratified by study design further verified the significant relationship between LIPI and PFS (Figure 2B).

### Subgroup analysis for OS

**FIGURE 3:**
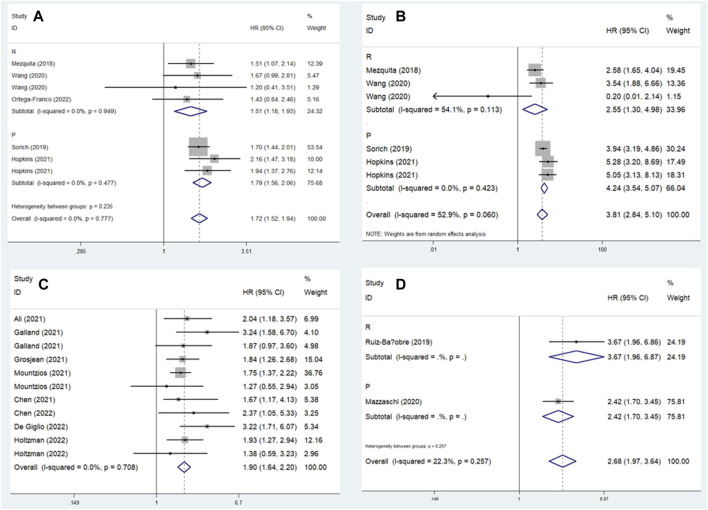
Subgroup analysis based on study design for the association between LIPI and overall survival of non-small cell lung cancer patients receiving immune checkpoint inhibitors. **(A)** LIPI 1 vs. 0; **(B)** LIPI 2 vs. 0; **(C)** LIPI 1–2 vs. 0; **(D)** LIPI 2 vs. 1 vs. 0.

In this meta-analysis, we conducted a subgroup analysis based on a comparison of the LIPI and study design. The pooled results further demonstrated that elevated LIPI was significantly related to worse OS (LIPI 1 vs. 0: HR = 1.72, 95% CI: 1.52–1.94, *p* < 0.001; LIPI 2 vs. 0: HR = 3.81, 95% CI: 2.84–5.10, *p* < 0.001; LIPI 1–2 vs. 0: HR = 1.90, 95% CI: 1.64–2.20, *p* < 0.001; LIPI 2 vs. 1 vs. 0: HR = 2.68, 95% CI: 1.97–3.64, *p* < 0.001; LIPI 2 vs. 0–1: HR = 2.75, 95% CI: 1.48–5.11, *p* < 0.001; LIPI 2 vs. 1: HR = 1.69, 95% CI: 1.37–2.08, *p* < 0.001). In addition, a more specific subgroup analysis based on study design for the comparison of LIPI 1 vs. 0 (Figure 3A), LIPI 2 vs. 0 (Figure 3B), LIPI 1–2 vs. 0 (Figure 3C), and LIPI 2 vs. 1 vs. 0 (Figure 3D) further identified the above findings. Detailed results are presented in Table 2.

**TABLE 2:** Results of meta-analysis.

	No. of studies	Hazard ratio	95% confidence interval	*p*-Value	I^2^ (%)	*p*-Value
Overall survival						
Overall	17	2.50	2.09–2.99	<0.001	68.6	<0.001
Retrospective	13	2.09	1.79–2.44	<0.001	23.5	0.182
Prospective	4	3.59	2.79–4.62	<0.001	69.2	0.011
LIPI 1 vs. 0	5	1.72	1.52–1.94	<0.001	0.0	0.777
Retrospective	3	1.51	1.18–1.93	0.001	0.0	0.949
Prospective	2	1.79	1.56–2.06	<0.001	0.0	0.477
LIPI 2 vs. 0	4	3.81	2.84–5.10	<0.001	52.9	0.423
Retrospective	2	2.55	1.30–4.98	0.006	54.1	0.113
Prospective	2	4.24	3.54–5.07	<0.001	0.0	0.423
LIPI 1–2 vs. 0	8	1.90	1.64–2.20	<0.001	0.0	0.708
Retrospective	8	1.90	1.64–2.20	<0.001	0.0	0.708
LIPI 2 vs. 1 vs. 0	2	2.68	1.97–3.64	<0.001	22.3	0.257
Retrospective	1	3.67	1.96–6.87	<0.001	—	—
Prospective	1	2.42	1.70–3.45	<0.001	—	—
LIPI 2 vs. 0–1	1	2.75	1.48–5.11	<0.001	—	—
Retrospective	1	2.75	1.48–5.11	<0.001	—	—
LIPI 2 vs. 1	1	1.69	1.37–2.08	<0.001	—	—
Prospective	1	1.69	1.37–2.08	<0.001	—	—
Progression-free survival						
Overall	18	1.77	1.64–1.91	<0.001	48.3	0.006
Retrospective	14	1.62	1.45–1.81	<0.001	34.4	0.081
Prospective	4	1.93	1.73–2.15	<0.001	64.8	0.023
LIPI 1 vs. 0	6	1.44	1.31–1.57	<0.001	0.0	0.979
Retrospective	4	1.35	1.13–1.61	0.001	0.0	0.927
Prospective	2	1.47	1.32–1.63	<0.001	0.0	0.982
LIPI 2 vs. 0	6	1.91	1.69–2.16	<0.001	41.0	0.105
Retrospective	4	1.75	1.36–2.24	<0.001	0.0	0.529
Prospective	2	1.97	1.71–2.27	<0.001	75.0	0.018
LIPI 1–2 vs. 0	6	1.60	1.26–2.04	<0.001	55.3	0.028
Retrospective	6	1.60	1.26–2.04	<0.001	55.3	0.028
LIPI 2 vs. 1 vs. 0	2	1.94	1.24–3.05	0.004	61.8	0.106
Retrospective	1	1.49	0.94–2.37	0.092	—	—
Prospective	1	2.37	1.73–3.25	<0.001	—	—
LIPI 2 vs. 0–1	2	2.22	1.47–3.36	<0.001	19.9	0.264
Retrospective	2	2.22	1.47–3.36	<0.001	19.9	0.264
LIPI 2 vs. 1	2	1.25	1.02–1.53		0.0	0.652
Retrospective	1	1.09	0.58–2.04	0.788	—	—
Prospective	1	1.27	1.03–1.57	0.027	—	—

LIPI: lung immune prognostic index.

### Subgroup analysis for PFS

**FIGURE 4:**
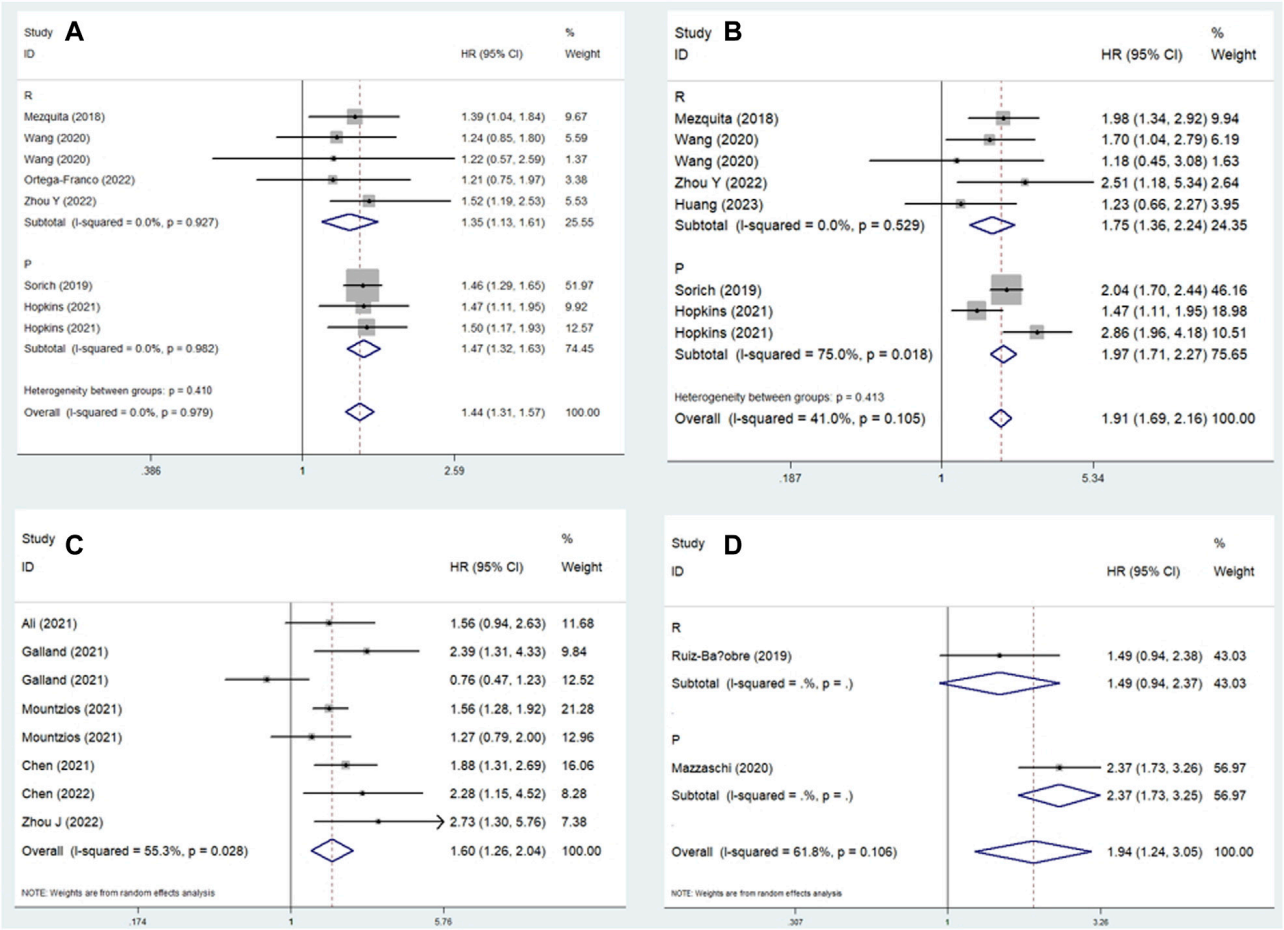
Subgroup analysis based on study design for the association between LIPI and progression-free survival of non-small cell lung cancer patients receiving immune checkpoint inhibitors. **(A)** LIPI 1 vs. 0; **(B)** LIPI 2 vs. 0; **(C)** LIPI 1–2 vs. 0; **(D)** LIPI 2 vs. 1 vs. 0.

Similarly, subgroup analysis for PFS based on the comparison of LIPI and the study design was performed. Pooled results revealed that elevated LIPI was obviously associated with poorer PFS (LIPI 1 vs. 0: HR = 1.44, 95% CI: 1.31–1.57, *p* < 0.001; LIPI 2 vs. 0: HR = 1.91, 95% CI: 1.69–2.16, *p* < 0.001; LIPI 1–2 vs. 0: HR = 1.60, 95% CI: 1.26–2.04, *p* < 0.001; LIPI 2 vs. 1 vs. 0: HR = 1.94, 95% CI: 1.24–3.05, *p* = 0.004; LIPI 2 vs. 0–1: HR = 2.22, 95% CI: 1.47–3.36, *p* < 0.001; LIPI 2 vs. 1: HR = 1.25, 95% CI: 1.02–1.53, *p* = 0.030). Furthermore, specific subgroup analysis based on study design for the comparison of LIPI 1 vs. 0 (Figure 4A), LIPI 2 vs. 0 (Figure 4B), LIPI 1–2 vs. 0 (Figure 4C), and LIPI 2 vs. 1 vs. 0 (Figure 4D) further confirmed the above findings. The detailed results are presented in Table 2.

### Sensitivity analysis

**FIGURE 5:**
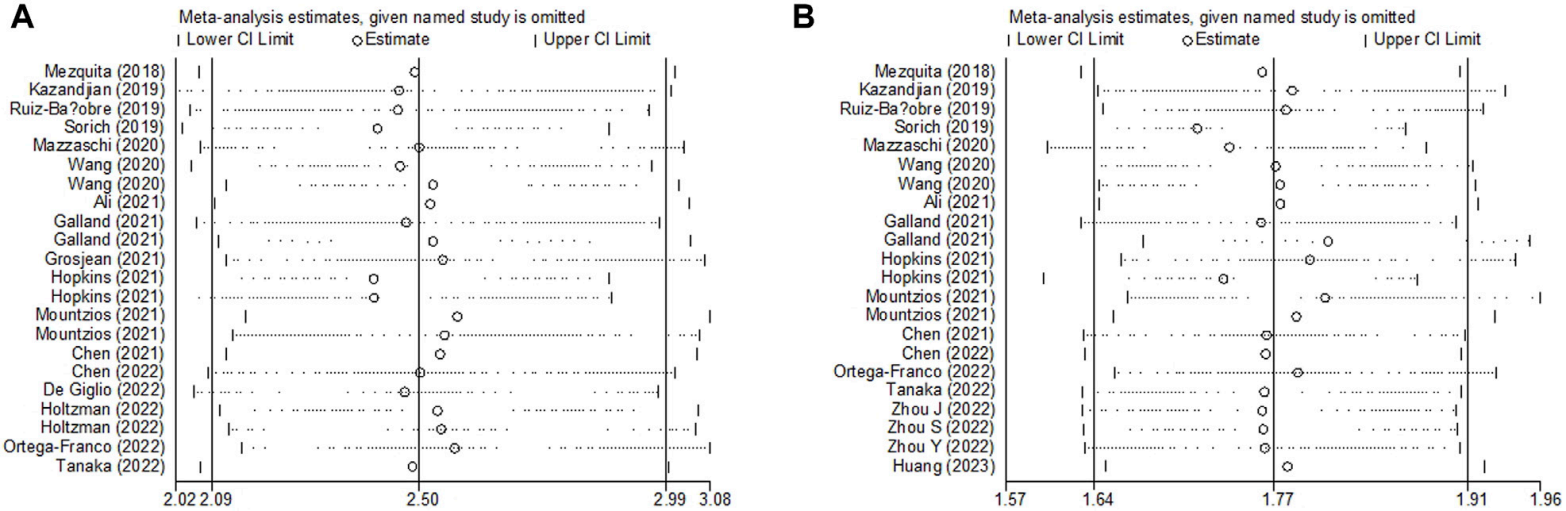
Sensitivity analysis about the association between LIPI and overall survival **(A)** and progression-free survival **(B)** of non-small cell lung cancer patients receiving immune checkpoint inhibitors.

Sensitivity analysis for the association between LIPI and OS and PFS was performed, which demonstrated that the pooled results of this meta-analysis were stable, and none of the included studies had an obvious impact on the relationship between LIPI and OS (Figure 5A) and PFS (Figure 5B) among immunotherapy-treated NSCLC patients.

### Publication bias

**FIGURE 6:**
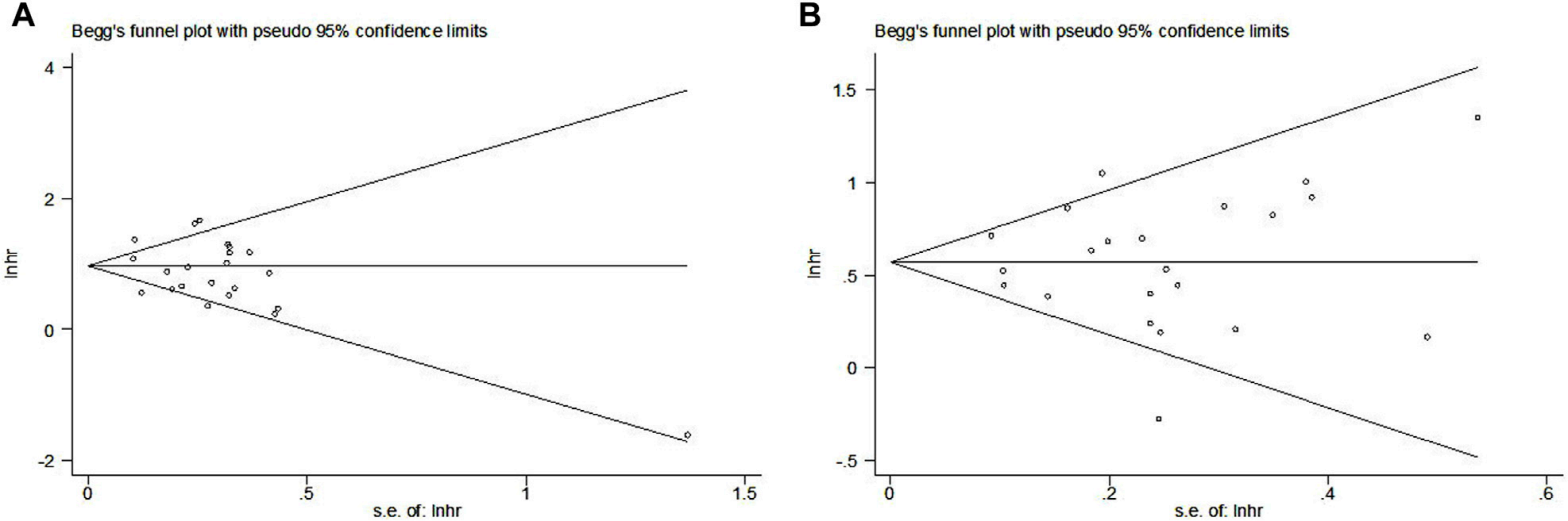
Begg’s funnel plots for the association between LIPI and overall survival **(A)** and progression-free survival **(B)** of non-small cell lung cancer patients receiving immune checkpoint inhibitors.

The Begg’s funnel plots for OS (Figure 6A) and PFS (Figure 6B) were both symmetrical, and the *p*-values of Egger’s test for OS and PFS were 0.208 and 0.992, respectively. Thus, no significant publication bias was observed in this meta-analysis.

## Discussion

This meta-analysis explored the predictive role of LIPI for the long-term survival of NSCLC patients who received ICIs based on current evidence, and the pooled results showed that LIPI was significantly associated with OS and PFS in this group of patients. Patients with elevated LIPIs were more likely to have a worse prognosis than patients with good LIPIs. Therefore, the LIPI could serve as a novel and reliable prognostic indicator in patients with NSCLC receiving ICIs.

The invasiveness of malignant tumors depends on the nature of tumor cells and their microenvironment. Previous studies have indicated that inflammation is a recognized feature of cancer, and inflammatory reactions play a crucial role in the process of carcinogenesis [40]. On the one hand, in malignant solid tumors, inflammatory stimulation leads to immune cell infiltration, angiogenesis, and fibroblast proliferation [41, 42]. In contrast, it is one of the mechanisms of immune tolerance, promoting tumor growth and dissemination, and activating oncogenic signaling pathways in cancer patients [43]. The dNLR was calculated using the neutrophil and lymphocyte counts. Neutrophils are key participants in tumor inflammation and immunity, and participate in tumor progression. Studies have found that neutrophils can produce vascular endothelial growth factor (VEGF), which plays an important role in mediating tumor angiogenesis and is a powerful immunosuppressive factor of natural and adaptive anti-tumor immunity [44]. In addition, neutrophil-derived proteases can degrade cytokines and chemokines and reshape the extracellular matrix, and neutrophil elastase in tumor cells can overactivate the PI3K pathway, further accelerating uncontrolled tumor proliferation [45]. It has been reported that T cells producing interleukin (IL)-17 can release CXC chemokines to supplement neutrophils, and IL17a is involved in resistance to ICIs [46]. Therefore, higher dNLR levels may reflect negative inflammation and contribute to resistance to ICIs. Peripheral blood lymphocyte count is considered a predictive factor for the prognosis of various cancers [47]. Lymphocytes play an important role in tumor-related immunity, have potential anti-tumor immune functions to inhibit tumor development, participate in cytotoxic cell death and cytokine production, and inhibit tumor cell proliferation and metastasis through the immune response to cancer [48].

LDH is widely distributed in major human organs and catalyzes the conversion of lactate and pyruvate. It is an indicator of tumor burden, cell damage, and necrosis. Studies have shown that elevated LDH levels are an adverse prognostic factor for tumors [49, 50]. Elevated LDH levels are a product of enhanced tumor glycolysis and hypoxia-induced tumor necrosis [51]. On one hand, in tumors with increased glycolytic activity, both aerobic and anaerobic glycolysis under hypoxia can affect immune cell function due to glucose deficiency or tumor acidity [52]. Furthermore, hypoxia itself or the excessive expression of hypoxia-regulated factors in highly glycolytic tumors may affect antitumor immunity [53]. In addition, the main switch for hypoxia-induced angiogenesis, hypoxia-inducible factor-1 (HIF-1), is activated by hypoxia and upregulates VEGF in tumors [54]. VEGF promotes tumor angiogenesis by inducing the proliferation and survival of endothelial cells, forming a large number of malformed and dysfunctional neovasculatures in the tumor [55]. These tumor blood vessels interfere with the active anticancer immune system and inhibit the therapeutic effect of ICI treatment. Therefore, LDH levels can affect the efficacy of ICIs.

Liu et al. included 12 studies with 4,883 solid cancer patients who received ICIs treatment and demonstrated that elevated pretreatment LIPI was significantly associated with worse OS (HR = 3.33, 95% CI:2.64–4.21, *p* < 0.001; HR = 1.71, 95%CI 1.43–2.04, *p* < 0.001) and PFS (HR = 2.73, 95% CI:2.00–3.73, *p* < 0.001; HR = 1.43, 95%CI 1.28–1.61, *p* < 0.001) [56]. However, only six studies explored the relationship between pretreatment LIPI and survival, and five studies were included in the pooled analysis [56]. In another meta-analysis by Xie et al., four studies involving 7,373 advanced NSCLC patients receiving ICIs, targeted therapy, or chemotherapy and their results revealed that intermediate and poor LIPI predicted worse OS (HR = 1.61, 95% CI:1.48–1.75, *p* < 0.01; HR = 2.74; 95% CI:2.26–3.33, *p* < 0.01) [57]. However, only three of the included studies identified the predictive role of pretreatment LIPI for OS in immunotherapy-treated NSCLC patients. Therefore, we performed this meta-analysis to determine the predictive value of LIPI for prognosis among patients with NSCLC receiving ICIs, and the pooled results indicated that LIPI could serve as a reliable prognostic factor in this group of patients.

This meta-analysis had several limitations that should be noted. First, all included studies were observational, and most of them were retrospectively conducted. Second, some of the included studies had relatively small sample sizes, which might have caused bias. Third, we were unable to conduct more subgroup analyses based on other parameters such as the pathological subtype, drugs of ICIs, and combinations of other therapies due to the lack of original data and sufficient information reported in the included studies.

## Conclusion

Overall, LIPI could serve as a novel and reliable prognostic factor in NSCLC treated with ICIs, and intermediate LIPIs predict a worse prognosis. However, further high-quality studies are required to verify our findings.

## Data Availability

The original contributions presented in the study are included in the article/supplementary material, further inquiries can be directed to the corresponding author.
